# RNA Binding Protein/RNA Element Interactions and the Control of Translation

**DOI:** 10.2174/138920312801619475

**Published:** 2012-06

**Authors:** Xavier Pichon, Lindsay A Wilson, Mark Stoneley, Amandine Bastide, Helen A King, Joanna Somers, Anne E Willis

**Affiliations:** Medical Research Council Toxicology Unit, Lancaster Rd, Leicester LE1 9HN UK

**Keywords:** Translation, 5’ untranslated region, IRES, uORF, TOP, polyA tail.

## Abstract

A growing body of work demonstrates the importance of post-transcriptional control, in particular translation initiation, in the overall regulation of gene expression. Here we focus on the contribution of regulatory elements within the 5’ and 3’ untranslated regions of mRNA to gene expression in eukaryotic cells including terminal oligopyrimidine tracts, internal ribosome entry segments, upstream open reading frames and cytoplasmic polyadenylation elements. These mRNA regulatory elements may adopt complex secondary structures and/or contain sequence motifs that allow their interaction with a variety of regulatory proteins, RNAs and RNA binding proteins, particularly hnRNPs. The resulting interactions are context-sensitive, and provide cells with a sensitive and fast response to cellular signals such as hormone exposure or cytotoxic stress. Importantly, an increasing number of diseases have been identified, particularly cancers and those associated with neurodegeneration, which originate either from mutation of these regulatory motifs, or from deregulation of their cognate binding partners.

## INTRODUCTION

**Fig. (1):**
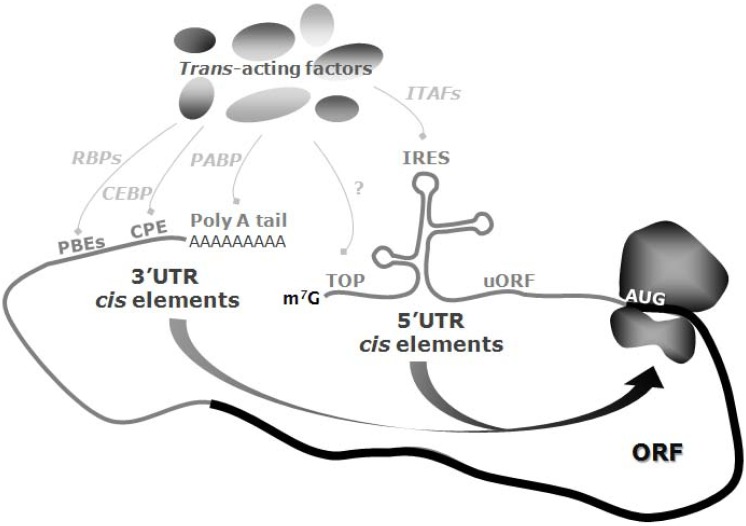
Schematic representation of translation initiation regulation through cis elements and trans-acting factors acting in the mRNA 5’ UTR and 3’ UTR. Regulation through the 5’ UTR can occur via the 5’-Terminal Oligopyrimidine tracts (TOP) motif, Internal Ribosome Entry Sites (IRESs) and their IRES-trans-acting factors and upstream Open Reading Frames (uORFs). Regulation through the 3’ UTR can occur via the interaction of RNA-binding proteins (RBPs) with Protein-binding elements (PBEs), the interaction of the cytoplasmic polyadenylation element-binding-protein (CPEBP) with cytoplasmic polyadenylation element (CPE) and also via the interaction of poly(A)-binding protein (PABP) with the poly(A) tail, which leads to mRNA circularisation.

Post-transcriptional control at the level of translation is an important mechanism by which gene expression can be regulated [1]. This process can act to modulate both global changes to translational rates and for selective subsets of mRNAs. Indeed, the data suggest that over 90% of all messages are subject to translational control and that this may be the predominant determinant of protein abundance in some cell types [1, 2]. Protein synthesis in eukaryotes can be considered to be a three-stage process (initiation, elongation and termination) and the majority of translational regulation occurs at the level of initiation, which is thought to be the rate-limiting step (discussed elsewhere). Initiation is a highly integrated process that requires canonical initiation factors (eIFs) and sequence elements within the 5’ and 3’ untranslated regions (UTRs) of the mRNA which can act in concert to regulate initiation [3], together with *trans*-acting proteins and RNAs [3, 4]. For many mRNAs initiation of translation occurs by a mechanism that has been termed cap-dependent scanning, which requires the binding of the trimeric complex eIF4F (which is comprised of eIF4E, the cap-binding protein, eIF4A a Dead box helicase, and eIF4G a large scaffold protein which contains binding sites for both eIF4E and eIF4A) to the 7-methyl G cap structure, followed by ribosome scanning to the first AUG codon positioned within a good context [5, 6]. This process is controlled by regulating the bioavailability of eIF4E which can be sequestered by its binding partners the 4EBPs, and by modifying the level of ternary complex (comprised of eIF2, tRNA_imet_ and GTP) which is required to bring the methionyl tRNA to the ribosome [6]. However, alternative methods to initiate translation in mammalian cells have been described which require RNA motifs and specific interacting proteins that directly influence the translation of individual mRNAs. These include in the 5’ untranslated region (UTR) TOP-motifs, internal ribosome entry segments, upstream open reading frames and in the 3’ UTR a number of unique elements in addition to the poly a tail length (Fig. **1**). It is known that micro-RNAs, which are short 21-23 nucleotide non-coding mRNAs, play a major role in post-transcriptional regulation of gene expression. These function by binding to 3’ UTR target sequences in mRNAs and negatively regulating the synthesis of the corresponding proteins. However the subject of microRNAs and their interaction with mRNA and RNA-binding proteins has been reviewed extensively elsewhere in great depth and is therefore not included here [7].

## TERMINAL OLIGOPYRIMIDINE TRACTS (TOPs)

In actively growing mammalian cells 30% of all translation involves a group of co-regulated mRNAs that possess a TOP motif in their 5’UTR. In these mRNAs the first base that proceeds the 7-methyl G cap is cytosine residue which is followed by a 5’ oligopyrimidine tract of 7-14 residues that forms the 5’ TOP motif [8]. For the majority of these mRNAs a TCT motif is also required for their transcription [9].

TOP containing transcripts encode ribosomal proteins and components of the translation apparatus including elongation factors, poly A binding protein and some subunits of the initiation factor eIF3 [10, 11]. Due to the substantial proportion of total cellular energy dedicated to synthesis of new ribosomes during cell growth and proliferation, it is essential for cell viability that protein synthesis initiated from TOP-containing mRNA is co-ordinately regulated [8, 12]. Indeed, even small perturbations in the relative levels of one of those proteins can result in cell cycle block, apoptosis, or if unchecked can lead to inappropriate cell division and tumorigenesis (discussed below).

It is thought that the presence of the 5’ TOP sequence element permits a unique method of translation initiation of this subset of mRNAs. For example, when cytoplasmic extracts are fractionated on sucrose density gradients to separate the sub-polysomal fractions (non-translating mRNAs) from the polysomes (translating mRNAs) those that contain the TOP-motif certainly appear to display a binary “all or none” association with ribosomes that is dependent on the growth state of the cell. It should however be noted that many of the mRNAs that contain TOP-motifs are short in total length such that only a few ribosomes are likely to be able to bind at once, making it harder to differentiate between active and inactive messages on sucrose density gradients. Nevertheless, TOP-containing mRNAs do appear to be particularly sensitive to the cell state when compared to other mRNAs e.g. actin [13]. For example, following cell stimulation (e.g. by growth factors), TOP-containing mRNAs are nearly fully loaded with ribosomes [10]. In contrast, during conditions of cell stress (e.g. serum starvation, apoptosis, UV exposure) which are accompanied by growth arrest, this group of mRNAs relocate to the subpolysomal region of the gradients [10, 14].

Despite many studies, the mechanisms by which 5’ TOP mRNA are translationally co-regulated remain poorly understood. Translation activation of 5′ TOP-containing mRNAs is known to require signalling through the mTOR pathway, but contradictory data indicate that other signalling pathways for example the PI3K/Akt signalling pathways may also be involved [15-17]. In addition, they may have different requirements for the eIF4F complex for ribosome recruitment when compared to other non-TOP-containing mRNAs. The first nucleotide that follows the cap structure lies within the binding pocket of the cap-binding protein eIF4E, and modelling studies strongly suggest different molecular interactions of purines compared to pyrimidines at this position [18, 19]. Therefore one would predict that TOP-containing mRNAs would interact differently with eIF4E from other capped messages.

It is highly likely that TOP-containing mRNAs require defined sets of *trans*-acting factors to allow their recruitment to the ribosome. Although some early data suggested that specific *trans*-acting factors control the association of TOP-containing mRNAs with the ribosomes (both positively and negatively [20, 21]), the proteins required and mechanisms used to achieve coordinated control of their expression have not been fully defined. Several *trans*-acting factors have been proposed to regulate the translation of 5’ TOP mRNA including pyrimidine-binding proteins [8]. For example, polypyrimidine tract binding protein (PTB) which is known to interact with other proteins, including hnRNP K or hnRNP E1, to initiate translation [22] may be involved in the activation of translation of 5’ TOP mRNAs by directly binding to the oligopyrimidine tract. In addition, several putative mRNA binding proteins have been identified which could modify TOP-mRNA translation either positively or negatively including the La antigen (La), cellular nucleic acid binding protein (CNBP) [23, 24], hnRPD/AUF1 [25] and TIAR-TIA1 [26]. TIAR and TIA1 are stress granule associated proteins, which, upon amino acid starvation, bind specifically to the 5’ end of the 5’TOP mRNAs [26] and cause inhibition of translation initiation, polysomal dissociation and relocalization of the 5’TOP mRNAs into the stress granules. This action is dependent upon both GCN2 activation and mTOR inactivation pathways [26].

Interestingly, it has been shown recently that RPS6 associates directly with mRNAs that contain a 5′ TOP motif leading to the translational inhibition of this subset of mRNAs [27]. It was suggested that RPS6-modulated expression of ribosomal proteins by the 5’ TOP motif was necessary for the correct stoichiometry of ribosomal RNAs and ribosomal proteins to allow the formation of active ribosomes [27]. In addition to *trans*-acting protein factors, there is also evidence to suggest that TOP-containing mRNAs are controlled by RNA-RNA interactions. For example, microRNA 10a has been shown to bind to a sequence downstream from the 5′ TOP element and acts to enhance the translation of a number of ribosomal protein transcripts mRNAs [28].

## INTERNAL RIBOSOME ENTRY SEGMENTS

An internal ribosome entry site (IRES) is a sequence or set of structural motifs within the 5’ UTR of a message that function to recruit the ribosome, independently of interactions with the mRNA 5’ 7-methyl G cap. This confers a translational advantage on the IRES-containing mRNAs during conditions where cap-dependent translation is inhibited. IRES elements were originally identified in the RNA genomes of two *picornaviridae*; poliovirus (PV [29]) and encephalomyocarditis virus (EMCV [30]). The RNA genomes of these *viridae* are naturally uncapped when present in the cytoplasm of the host cell, yet they are efficiently translated. During viral infection cap-dependent translation is reduced due to the modification of canonical initiation factors e.g. by viral protease-mediated cleavage of eIF4G, which provides a selective advantage of translation from the IRES containing viral transcripts.

Cap-dependent translation of eukaryotic mRNAs is inhibited during periods of cellular stress, apoptosis and also during certain stages of the cell cycle. Accordingly, cellular IRES-containing mRNAs encode for proteins important for cellular fate decisions, such as pro- and anti-apoptotic proteins, cell cycle proteins and transcription factors. The first cellular IRES element was identified in the 5’ UTR of the mRNA encoding the protein chaperone BiP [31]. This IRES was shown to permit the translation of BiP during poliovirus infection where eIF4G is cleaved and cap-dependent translational rates are diminished. In order to identify mRNAs that can be translated in a cap-independent manner in response to a specific stress stimuli, a number of studies have used sucrose density gradients and polysomal profiling [32-34]. The presence of putative IRESs in the 5’ UTRs of mRNAs that remain polysomally associated under such conditions are then validated using *in vitro* reporter assays, in particular by using a dicistronic reporter assay which involves the use of a construct encoding two reporter proteins separated by an intercistronic spacer region. The putative IRES element is cloned into this spacer region, such that any product translated from the first cistron is produced in a cap-dependent manner, and any from the second cistron is produced in a cap-independent or IRES-dependent manner. The possibility of the presence of a cryptic promoter or splice site in the putative IRES or the occurrence of ribosomal read-through from the first ORF must then be eliminated using stringent control experiments. After addressing these issues studies have found that approximately 3-5% of profiled mRNAs contain IRES elements. Interestingly, the lists are largely distinct for each stress condition, indicating specific translational re-programming in response to different stress stimuli. Accordingly, current estimates suggest that around 10% of mRNAs in the human transcriptome are likely to contain IRES elements [35] (Table **1**). In support of these data an IRES motif containing (CCU)n repeats as part of a polypyrimidine tract was identified in the 5’ UTRs of approximately 10% of the transcriptome [36].

Regulation of IRES-mediated translation is undoubtedly complex, given that not every IRES is active during all conditions, nor at every stage in the cell cycle. The data suggest that both cellular and viral IRESs are regulated by specific IRES-*trans*-acting factors (ITAFs). ITAFs appear to function as RNA chaperons, remodelling IRES-RNA structure into a permissive structure for other ITAFs or the 40S ribosomal subunit to bind. For example, the pro-apoptotic APAF-1 IRES is first bound by the ITAF UNR, which then remodels the IRES secondary structure to reveal a binding site for the ITAF nPTB, subsequent association with nPTB permits the recruitment of the 40S ribosomal subunit [37]. The anti-apoptotic BAG-1 IRES was found to act in a similar fashion, with the ITAFs PCBP1 and PTB [38].

Of all ITAFs identified and characterised thus far, a large number of them belong to the heterogeneous nuclear ribonucleoprotein (hnRNP) family and hnRNPI (PTB), appears to be very important in this regard (Table **1**) [36]. Proteins in this family are highly abundant and many are able to shuttle between the nucleus, where they are predominantly located, to the cytoplasm. These proteins play a role in numerous steps of RNA metabolism including pre-mRNA splicing, 3’ end processing, nuclear export, mRNA localisation and control of IRES-mediated translation [39]. Thus, it is tempting to speculate that ITAFs required to regulate IRES-mediated translation are loaded onto the mRNA during transcription and splicing. Control of IRES-mediated translation could be exerted either via restricting the IRES-containing RNA to the nucleus until translation is required, at which point it is exported to the cytoplasm as a mRNP complex, or repressive ITAFs may bind the IRES-containing RNA in the cytoplasm until a release signal is received at which point they are replaced by shuttling stimulatory ITAFs. Cellular IRESs also require certain canonical initiation factors for function. For example, it has been shown that the N- and c-Myc IRESs require the central fragment of eIF4G, eIF4A and eIF3 but not the cap-binding protein eIF4E [40]. 

In addition to ITAFs, several recent studies have demonstrated that modified ribosomal RNA is required for translation for certain IRESs. For example, loss of functionality of the x-linked dyskerin gene (DKC1) which catalyses the isomerisation of specific uridines in 18S rRNA into pseudouridines was shown to significantly reduce IRES-mediated expression of both p53 [41] and p27 [42]. Loss of these essential cell cycle regulators results in dysregulation of the cell cycle, and unchecked oncogenic progression in X-linked dyskeratosis congenita (XDC) patients. In addition, rRNA methylation has been shown to be required for the activity of the c-*myc*, CAT-1, c-src and SNAT2 IRESs. Interestingly, inhibition of rRNA methylation only affected cellular IRESs and not viral IRESs [43]. These data suggest that viral and cellular IRES elements are functionally distinct in their mechanism of action, and also that different populations of ribosomes exist in the cell which are responsible for the translation of different subsets of mRNA, thus conferring yet another level of regulation over the energy intensive process of translational initiation.

## UPSTREAM OPEN READING FRAMES

According to the scanning model of cap-dependent translation initiation, after it is recruited to the cap-proximal of the mRNA region, the ribosome will initiate polypeptide synthesis at the first start codon it encounters. Thus it was originally assumed that for the majority of mRNAs, that this first AUG and the main protein coding sequence (MCS) start site would coincide [44]. Subsequent studies revealed that many mRNAs contain one or more AUG codons upstream of the MCS, often located in long and potentially structured 5’UTRs [45]. Such upstream AUGs (uAUGs) can be recognised by the mRNA translation apparatus and form the initiation site for an upstream open reading frame (uORF). In a simple scenario, translation initiation at a single uORF can constitutively inhibit the MCS translation. After translating the uORF, ribosomes terminate and must acquire additional factors, in particular the ternary complex (eIF2. tRNAmet.GTP), to re-initiate at the downstream MCS. Translation of the MCS is repressed if post-termination ribosomes do not become competent to re-initiate before they reach the MCS start codon. The intrinsic efficiency of uORF translation repression is governed by a number of factors, including recognition of the uORF start codon, the sequence context of the uORF termination codon, the uORF length, and the distance between the uORF and the MCS [46-49]. In particular, longer uORFs and shorter intercistronic distances decrease the re-initiation rate. Paradoxically, uORFs can also increase the translation rate of certain mRNAs under conditions of cell stress. The paradigm for this mechanism is the de-repression of *GCN4* mRNA translation in nutrient starved yeast cells, which is dependent on four uORFs. In non-starved cells, ribosomes translate uORF1 and subsequently, due to the presence of readily available ternary complex, re-initiate at uORFs [45-47]. Translation of these downstream uORFs ensures that re-initiation at the MCS start site is a rare occurrence. However, phosphorylation of eIF2α in starved cells limits the cellular levels of ternary complex, such that the ribosome scans through uORFs [45-47], and instead re-initiates at the *GCN4* start site. Thus a translational switch ensures that cells respond rapidly to amino acid deprivation and respond by initiating a compensating program of gene expression [46-48]. In mammals, cell stress simultaneously inhibits general translation and activates the integrated stress response (ISR), a pro-survival gene expression program. The ISR transcription factors ATF4 and ATF5 are induced by cell stress in a *GCN4*-like mechanism that involves two uORFs and phosphorylation of eIF2α [50-52]. Alternative stress-induced translation control mechanisms have also been reported that do not conform to the *GCN4* re-initiation paradigm. These mechanisms depend on one or more uORFs and reduced ternary complex, and in the case of the *Chop* and *Gadd34* mRNAs appear to involve the bypass of a single inhibitory uORF [53-55]. Another example in a separate stress pathway is the induction of the DNA damage response. Following UVB treatment DNAPK mediated eIF2α phosphorylation leads not only to a general translational inhibition but also the translational upregulation of a number of uORF containing DNA repair genes including *ERCC5, ERCC1* and* DDB1.* The presence of these uORFs were essential for the translation of the downstream cistrons, however the mechanism has yet to be fully realised as a simple reduction in cellular ternary complex levels alone proved not sufficient to explain this induction [54].

The importance of uORFs is underscored by recent bioinformatics analysis, which identified these elements in 35-49% of human and rodent genes and revealed a widespread reduction in the corresponding protein levels [56-58]. Approximately half of these transcripts contain a single uORF, and the remainder bear multiple uORFs with the potential to add to the complexity of the mRNA translation control mechanism. Furthermore considerable diversity has been noted in the number, position and length of uORFs. To date approximately 100 instances of translation control through uORFs have been documented [58]. The mechanisms detailed thus far include simple translation repression, *GCN4*-like de-repression and ribosome bypass. In addition, the uORF peptide can interfere with MCS translation [59]. Other uORFs can influence mRNA stability or trigger co-ordinated translation repression and nonsense-mediated decay [58, 60]. Finally translation of an uORF controls internal translation initiation on the cat-1 mRNA [61]. 

Overall it is clear that uORFs are a widely used element in the regulation of gene expression with the potential to mediate both simple and complex control mechanisms in different cellular contexts. Given the large number of transcript bearing these elements it is clear that the study of these elements still has much to inform us about their contribution to the control of gene expression and their contribution to the disease state. Moreover a detailed understanding of the mechanisms of uORF-mediated control may lead to novel therapeutic interventions. 

## REGULATION OF mRNA TRANSLATION VIA THE 3’UTR

The 3’UTR contribution to translation regulation involves a number of different mechanisms including poly(A) length, the cytoplasmic polyadenylation element (CPE), specific RNA binding elements and miRNAs (the latter are not discussed here). These can exert both general and message specific effects on translation.

Translation initiation is stimulated by the presence of a poly(A) tail. This enhancement is likely to arise by the interaction of PABP with both the poly(A) tail and the eIF4F complex [62], which exerts conformational changes on eIF4E [62, 63]. Furthermore, it has been suggested that the mRNA circularization that results from the PABP-eIF4G interaction may facilitate the re-initiation of ribosomes that have terminated their translation of the mRNA [64].

The length of the poly(A) tail is known to affect translational rates and mRNAs with short poly(A) tails (<50 A residues) are generally translationally repressed. Regulation of poly(A) tail length is a means of both global and specific translation control. It is critical during oogenesis, when specific mRNAs are stored in a translationally repressed state with a short poly(A) tail and then translationally activated by polyadenylation at specific stages of oocyte or early embryonic development [65]. During oocyte maturation, polyadenylation by the Cytoplasmic poly(A)-polymerase Gld2 occurs, resulting in a long poly(A) tail and translational stimulation via the closed loop mechanism described above [66]. 

Poly(A) length reduction is achieved by deadenylation. In somatic cells, deadenylation is linked to mRNA stability; removal of the poly(A) tail promotes decapping and exonuclease-mediated degradation [67]. However a recent study in mouse fibroblasts revealed the presence of a large population of mRNAs with a short or no poly(A) tail [68]. Since deadenylation and mRNA degradation may not be as tightly linked as originally suggested, translational regulation by poly(A)-tail length may play a more significant role in adult cells than was previously thought.

* Xenopus* oocytes have provided an excellent model in which to study control via the poly(A) tail. Regulation of translation by cytoplasmic polyadenylation is controlled by the CPE; a Uridine-rich sequence within the 3’UTR. The CPE is involved in both the activation [69] and repression of polyadenylation [70]. Activation of polyadenylation during oocyte maturation is facilitated by the binding of CPE-binding protein (CPEB) to the CPE within 3’UTR of the target mRNA. Through the binding of CPEB, several additional proteins including cleavage and polyadenylation specificity factor (CPSF), Gld-2 and the deadenylase poly(A)-specific ribonuclease (PARN) become associated with the target mRNA, forming a protein complex. As PARN is more active than Gld-2 in this complex, the poly(A) tail is short. However during oocyte maturation, phosphorylation at several sites on CPEB results in rearrangement of the complex and subsequent ejection of PARN, allowing elongation of the poly(A) tail by Gld2 and thus dormant mRNA becoming actively translated [66].

Two models have been proposed to account for the repression of translation through CPE. The first requires maskin, a CPEB binding protein [71] which also binds to eIF4E, via an eIF4G-like domain. This configuration of factors prevents the binding of eIF4G to eIF4E and inhibits the assembly of the 48S complex [69]. An alternative model proposes that PARN is recruited to the CPE and its proximity to the cap prevents eIF4E from binding, thus preventing initiation from occurring. The binding of maskin is not required in this model [66]. Translational regulation by CPEB polyadenylation can also require the RINGO/Spy protein. It has been shown that the reversible binding of Pumilio 2 to the Pumilio-Binding Elements (PBEs) in the 3’UTR of RINGO/Spy mRNA leads to the translational repression of this mRNA. Following oocyte maturation Pumilio 2 dissociates from the 3’UTR allowing the translation of RINGO/Spy mRNA and thus activation of CPEB polyadenylation [72].

There are also many 3’UTR regulatory elements that achieve regulation without modifying poly-(A) tail length and instead require the binding of a RNA-binding protein to a specific element in the 3’UTR. A well-described example in *Drosophila* is the translational repression of oskar mRNA by the RNA-binding protein Bruno. Bruno binds oskar mRNA at a specific sequence within its 3’UTR leading to the binding of the eIF4E-binding protein Cup to eIF4E. This results in the formation of a 5’-3’ interaction via the eIF4E-Cup-Bruno complex, thereby preventing interaction of eIF4E with eIF4G and thus translation initiation. This mechanism leads to an asymmetric distribution of Oskar protein in the oocyte [73].

Translation repression is also important for translation regulation of Caudal, a *Drosophila* embryonic protein. The RNA-binding protein Bicoid represses caudal mRNA by binding to a specific motif in its 3’UTR. As Bicoid also contains an eIF4E-binding motif, the resulting complex prevents eIF4E-eIF4G interactions and thus inhibits translation initiation [74]. An eIF4E-related cap binding protein (d4EHP) has been shown to bind Bicoid and also the 5’cap structure of caudal mRNA, also leading to an inhibition of translation initiation [75].

In erythroid precursor cells binding of heterogeneous nuclear ribonucleoprotein K (hnRNPK) and hnRNP E1 to the differentiation control element (DICE) in the 3’UTR of r15-LOX mRNA leads to its translational repression by inhibiting the joining of the 60S subunit [76, 77]. More recently the DEAD box RNA helicase family member DDX6 has been identified as a part of the repressive complex with hnRNP K/E 1-DICE in maintaining hr15-LOX mRNA silencing in premature cells [78].

## THE ROLE OF UTRs IN DISEASE

Here we provide examples of the roles of various 5’ and 3’ untranslated elements in disease, with the exception of microRNAs. 

## TERMINAL OLIGPYRIMIDINETRACT mRNAs (TOP mRNAs)

Since mRNAs containing TOPs usually code for ribosomal components and proteins with key roles in translation and growth, any mutations seriously affecting their function are likely to have severely deleterious effects and be selected against at an early stage in embryonic development. However changes to levels of expression of TOP-containing mRNAs are implicated in several diseases.

Ribosomal genes are implicated in Diamond-Blackfan anaemia (DBA) a congenital hypoplastic anaemia that presents at a very young age, with 25% of patients carrying mutations in small-ribosome component RPS19. RPS19 self-regulates its expression by binding to its own 5’ UTR TOP region, which leads to an increase of translation of the corresponding protein. However, the affinity of RPS19 binding for its own mRNA is reduced when mutations associated with DBA are introduced into the mRNA [79]. There are also data which suggest that RPS19 interacts with the TOP elements of other 40S ribosomal subunit mRNAs (in particular S20, S21 and S24) to promote their translation, with RPS19 reduction leading to widespread ribosomal abnormalities [80]. An imbalance between ribosome biogenesis and total mRNA levels can ultimately lead to stabilisation of the p53 pro-apoptotic protein: this may provide a positive selection pressure for the loss of p53 that could explain an increased risk of some leukaemias in DBA [81, 82]. 

Therapeutic strategies to target abnormal TOP mRNA translation may in future have the dual advantage of ameliorating disease symptoms whilst reducing the risk of p53 mutation-related diseases developing later. 

## IRESs

Translational dysregulation of protoocogenes makes a major contribution to the development of cancer. This can be mediated by mutations or polymorphisms in the RNA-binding elements that regulate the expression of this group of proteins, leading to up-regulated protein expression without a corresponding change in the level of the mRNA. For example, in multiple myeloma, an incurable cancer of abnormally proliferating plasma cells, there is dysregulated expression of c-Myc. While in some patients this arises from changes in the transcriptional level, a mutation within the c-Myc IRES, predicted to alter its secondary structure, is strongly over-represented in MM patients [83]. The mutation increases over 3-fold the rate of translation of c-Myc without changes to the underlying levels of mRNA. The mutant IRES shows greatly increased affinity for at least two of the ITAFs (PTB1 and Yb1) known to bind the c-Myc IRES element [84], and elevated expression of c-Myc in MM cell lines correlates with the expression of these proteins, rather than the levels of c-Myc mRNA [85]. Reduction of the ITAFs expression led to a decrease in rates of proliferation in MM-derived B-cell lines to a level comparable to control lines [85]. This finding is important because it suggests the possibility of therapeutic intervention in IRES-mediated diseases through targeting the expression of specific ITAFs. 

## uORFs

To date several examples of altered uORF-mediated translational repression that predispose individuals to a diseased state have been identified. 

Thyroid Peroxidase (TPO) mRNA contains seven uORFs that severely inhibit the translation of the MCS, and the most effective of these is uORF7. Three familial mutations have been identified in the TPO gene that directly affect the efficiency of uORF-mediated translation repression: a frameshift mutation in uORF7 that fuses this sequence in frame with the TPO coding region, a mutation that shortens uORF7, and a mutation that result in a TPO mRNA that entirely lacks uORF7. In each of these cases the inhibitory effect of uORF7 is reduced resulting in increased TPO protein levels and as a result Hereditary Thrombocythemia [86]. In an analogous example, a mutation in an uORF in the mRNA encoding the BACE-1 protein (required for maturation of the Alzheimer’s Disease (AD)-associated Amyloid Precursor Protein (APP) into Amyloid β) is present in some patients with AD [87]. As a consequence the normal uORF-dependent inhibition of BACE-1 translation is prevented [88, 89] leading to translation up-regulation of the corresponding protein.

Acquisition of an uORF can also pre-dispose individuals to the diseased state. In some melanoma patients a single point mutation introduces an uORF into the *cdkn2a* 5’UTR. The mutation reduces the synthesis of the tumour suppressor protein p16^*ink4a*^ contributing to the development of the malignant state [90]. More recently a systematic screen of the Human Genome Disease Database for mutations that eliminate or introduce uORFs in disease-associated mRNAs revealed 11 novel mutations that were present only in affected patients. In five of these conditions, Gonadal dysgenesis (*SRY*), Van der Woude syndrome (*IRF6*), Carney complex type 1 (*PRKAR1A*), Hereditary pancreatitis (*SPINK1*), and Thalassaemia-β (*HBB*), the uORFs substantially reduced translation at the genuine initiation codon and most likely contribute to disease pre-disposition [58]. In addition, single nucleotide polymorphisms (SNPs) that create or delete uORFs have been identified in more than 500 mRNAs in humans [58]. Thus variation in uORF-mediated translation control could account for differences in individual phenotype, the response to cellular conditions and predisposition to specific conditions.

## AU-RICH ELEMENTS (AREs)

AU-rich elements are frequently found within the 3’ UTRs of mRNAs coding for proteins with roles in the immune system, growth and survival. Dysregulation of ARE-binding proteins (AUBPs) can lead to disease (for reviews, see [91] and [92]). Diverse functions and interactions mean AUBPs can have pro- and anti-oncogenic effects in different contexts. The anti-apoptotic protein Bcl-2 is frequently overexpressed in cancer, but AUF1 promotes its degradation through recruitment of the mRNA to the exosome [93]. AUF1 competes for Bcl-2 binding with another AUBP, nucleolin, which protects Bcl-2 mRNA from exosomal decay [94]. In anaplastic large cell lymphoma (ALCL), AUBPs AUF1 and HuR co-localise with the fusion protein Nucleophosmin-Anaplastic Lymphoma Kinase (NMP-ALK) and are subsequently phosphorylated. This may result in the AUBPs stabilising target mRNAs with a role in tumorigenesis, including c-Myc, c-Jun and c-EBPβ [95].

## INVOLVEMENT OF MULTIPLE MECHANISMS OF mRNA CONTROL IN DISEASE

As discussed above deregulation of translation is frequently implicated in diseases in which aberrant protein expression does not correlate with mRNA levels. However, teasing apart which mechanisms of translational control contribute to disease development can be challenging, since mRNAs frequently contain several (sometimes overlapping) control elements, with multiple interacting factors whose own status may also be perturbed. 

One example of such complexity in translational control is the Vascular Endothelial Growth Factor A (VEGF-A) protein, which is upregulated in many diseases such as cancers, arthritis, diabetic retinopathy and psoriasis. Complex transcriptional control and alternative splicing generate at least 9 transcript variants containing long 5’ and 3’ UTRs. Within the 5’ UTR there are three alternative CUG start codons: translation from two of these is influenced by proximity to one of two IRESs. An uORF lies within one of the IRESs, where its cap-independent translation acts to negatively regulate a more diffusible (and more potently tumorigenic) isoform [96]. Additionally the 3’ UTR contains many AU-rich elements and many alternative polyadenylation signals. Her-2 mRNA provides another example of translational regulation by a number of individual elements. Under normal conditions, a uORF within the 5’ UTR of the Her-2 receptor negatively regulates translation. However, overexpression of Her-2 in breast cancer cells appears to result from de-repression elements within the 3’ UTR, together with their cognate binding factors, interacting with the 5’ UTR to promote reinitiation at the Her-2 AUG [97]. 

## SUMMARY

In the last decade, huge advances have been made in our knowledge of translation control and it is now understood to be the most significant factor determining protein abundance in at least some cell types [2]. 

While control of transcription and protein turnover are necessarily also of fundamental importance, translation control can augment transcriptional variation with a more nuanced temporal “fine tuning” of expression levels, in addition to allowing spatial intracellular localisation of expression; the latter is especially critical in the context of oocytes and cells lying at the surface of epithelia, tissues and organs. Controlling translation also allows an almost instant response to signals such as hormones or stress, and can thereby reduce the overall cellular energy requirements, were protein turnover the only means of post-transcriptional control.

The control elements within the 5’ and 3’ UTRs of mRNAs described here play essential roles in translation control; this can be exerted at the level of an individual message or affect a large group of transcripts. Taken together, and with miRNAs, they allow for an almost limitless range of control possibilities and can be co-ordinated within a single mRNA to provide expression control. Future research into the UTRs, particularly through increased use of deep-sequencing based ribosome profiling [119] is likely to reveal yet more subtlety, in addition to new control elements and more interacting protein and RNA partners. A growing number of diseases stand to become linked with such elements as we correlate their occurrence with polymorphisms within the UTRs. It is also likely that breakthroughs will be made in understanding how different elements physically act to enhance or repress translation, an aspect currently poorly understood. Whether global paradigms will emerge, or whether there will be almost as many modes of action as there are transcripts, remains to be seen.

## Figures and Tables

**Table 1.** A List of Cellular IRES-Containing mRNAs, the Function of the Protein they Encode, and the ITAFs Identified to Date as Important for Their Activity

Cellular IRES-Containing mRNA	Encoded Protein Function	Identified ITAFs	References
Apaf-1	Pro-apoptotic	DAP5, hnRNPA1, PTB, UNR	[98]
Bag-1	Anti-apoptotic	PCBP1, PTB	[99]
Bcl-2	Anti-apoptotic	DAP5	[100]
BiP	ER protein chaperone	hnRNPQ, PTB	[31]
Cat-1	Amino acid transporter	hnRNPL, PTB	[101]
C-*myc*	Transcription factor	hnRNPA1, PCBP1, PCBP2, hnRNPK, GRSF1, NONO, PSF, PTB, YB1	[102, 103]
CDK1	Cell cycle control	DAP5	[104]
Cyclin D1	Cell cycle control	hnRNPA1	[105]
Cyclin T1	Cell cycle control	PTB	[32]
DAP5	Translation initiation	DAP5	[106]
FGF2	Growth factor	hnRNPA1	[107]
Hiap2	Anti-apoptotic	DAP5	[108]
HIF-1α	Transcription factor	PTB	[109]
IGFR	Growth factor receptor	PTB	[40]
Mnt	Transcription repressor	PTB	[110]
MTG8a	Transcription factor	PTB	[36]
p27 Kip1	Cell cycle control	PTB	[111]
p53	DNA damage response	PTB	[112, 113]
PDGF/c-*sis*	Growth factor	hnRNPC1/C2	[114]
PITSLRE	Cell cycle control	UNR	[115]
Rev-erbα	Transcription repressor	hnRNPQ, PTB	[116]
UNR	RNA stability, ITAF	hnRNPC1/C2, PTB, UNR	[117]
XIAP	Anti-apoptotic	hnRNPA1, hnRNPC1/C2, HuR, La, mdm2, PTB	[118]

Alternative names: PTB (hnRNPI), PCBP1 (hnRNPE1), PCBP2 (hnRNPE2). Abbreviations: heterogeneous nuclear ribonucleoprotein (hnRNP), PTB (polypyrimidine tract binding protein), UNR (upstream of N-ras), PCBP (poly (rC) binding protein), GRSF (G-rich RNA sequence binding factor), NONO (non-POU domain containing), PSF (splicing factor proline/glutamine rich), YB1 (Y-box binding protein).
